# Transcription Factor Binding Site in Promoter Determines the Pattern of Plasmid-Based Transgene Expression In Vivo

**DOI:** 10.3390/pharmaceutics16040544

**Published:** 2024-04-15

**Authors:** Chunbo Zhang, Dexi Liu

**Affiliations:** Department of Pharmaceutical and Biomedical Sciences, University of Georgia College of Pharmacy, Athens, GA 30602, USA; cbzhang@ncu.edu.cn

**Keywords:** promoter, transcription factor binding site, gene expression regulation, gene therapy, nonviral vector, hydrodynamic gene delivery

## Abstract

Understanding the regulation of transgene expression is critical for the success of plasmid-based gene therapy and vaccine development. In this study, we used two sets of plasmid vectors containing secreted embryonic alkaline phosphatase or the mouse IL-10 gene as a reporter and investigated the role of promoter elements in regulating transgene expression in vivo. We demonstrated in mice that hydrodynamic transfer of plasmids with the CMV promoter resulted in a high level of reporter gene expression that declined rapidly over time. In contrast, when plasmids with albumin promoters were used, a lower but sustained gene expression pattern was observed. We also found that plasmids containing a shorter CMV promoter sequence with fewer transcription factor binding sites showed a decrease in the peak level of gene expression without changing the overall pattern of reporter gene expression. The replacement of regulatory elements in the CMV promoter with a single regulatory element of the albumin promoter changed the pattern of transient gene expression seen in the CMV promoter to a pattern of sustained gene expression identical to that of a full albumin promoter. ChIP analyses demonstrated an elevated binding of acetylated histones and TATA box-binding protein to the promoter carrying regulatory elements of the albumin promoter. These results suggest that the strength of a promoter is determined by the number of appropriate transcription factor binding sites, while gene expression persistence is determined by the presence of regulatory elements capable of recruiting epigenetic modifying complexes that make the promoter accessible for transcription. This study provides important insights into the mechanisms underlying gene expression regulation in vivo, which can be used to improve plasmid-based gene therapy and vaccine development.

## 1. Introduction

Plasmid-based vectors have been studied for their applications in gene therapy and vaccine development using nonviral delivery systems. A plasmid-based vector aims for high-level and persistent transgene expression. Different promoters have been previously investigated for their activity in driving gene expression. Viral promoters, such as cytomegalovirus immediate early promoter (CMV), the simian virus 40 (SV40) [1], Rouse sarcoma virus long terminal repeat (RSV) [2], and murine leukemia virus (MLV) [3] promoters, are the strongest in driving gene expression in mammalian cells [4,5]. However, some viral promoters are prone to silencing, resulting in transient transgene expression. Mammalian promoters have also been explored for plasmid-based transgene expression, including the elongation factor 1a (EF1a) promoter, the apoA1 promoter, the apoE promoter, the albumin promoter, the alpha-1 antitrypsin promoter, and others (for review, see [6]). Most of these promoters appear tissue-selective, exhibiting higher activity in one tissue but relatively low activity in others. With a few exceptions, the mammalian promoters are weaker than the viral promoters in driving transgene expression but exhibit better activity in sustained transgene expression.

Advances in understanding genomic gene expression in eukaryotes have generated a significant interest in identifying the genetic elements capable of regulating transgene expression and in developing chimeric promoters with desirable properties for plasmid-based applications. Earlier studies have shown that inclusion into a plasmid of chromatin insulator sequence [7,8], matrix attachment region [9], locus control region [10,11], intron sequences [12,13], or optimal sequences for transcription capping, splicing, or polyadenylation signal improves persistency of transgene expression in mice [6,14,15,16]. It was also shown that bacterial sequences in the plasmid negatively contribute to the longevity of transgene expression, and the removal of these sequences improves transgene expression in vivo [17,18]. In addition, efforts have also been made to prolong transgene expression by removing CpG sequences from plasmid vectors that are known to recognize toll-like receptors for immune activation and to cause gene silencing through DNA methylation [19,20]. Mechanistically, however, regulation of plasmid vector-based transgene expression in vivo is largely unknown and remains elusive.

For plasmid vector-based gene expression, plasmid DNA, when delivered to cells, enters the nucleus and interacts with histones. The ionic histone-DNA interaction commonly blocks the access of the transcription factors to the promoter region, resulting in a decrease in gene expression, and the transgene becomes silenced with time. We have shown that treatment of transfected animals with histone deacetylase inhibitors reactivates the silenced transgene [21], suggesting that the DNA in the histone-DNA complexes can be dissociated by histone acetylation. The purpose of the current study is to investigate how the transcription factor binding site in a promoter regulates transgene expression. The central hypothesis is that the pattern of transgene expression in vivo is determined by the number and type of transcription factor binding sites in a promoter.

To test this hypothesis, we employed two types of plasmid vectors. One contains an albumin promoter to drive low but sustained transgene expression, and the other has a CMV promoter to drive high but transient gene expression. A series of plasmids with deletions in the CMV promoter region were generated to identify the elements that are responsible for transient reporter gene expression. Additional plasmid vectors were created with all transcription factor binding sites in the CMV promoter replaced by those from an albumin promoter to examine the function of transcription factor binding sites in the albumin promoter. Our results showed that sustained transgene expression was achieved by replacing transcription factor binding sites in the CMV promoter with a single transcription factor binding site in the albumin promoter. The mechanistic study revealed that an elevated binding of acetylated histones to the transcription factor binding site in the albumin promoter is responsible for sustained transgene expression. These results demonstrate that a high number of transcription factor binding sites in the CMV promoter, while making CMV a strong promoter in mouse liver, is responsible for gene silencing. Prolonged transgene expression driven by an albumin promoter is due to its ability to recruit the acetylated histones to the promoter region. The results reveal the importance of using appropriate transcription factor binding sites for plasmid constructs to achieve desirable transgene expression for gene therapy studies.

## 2. Materials and Methods

### 2.1. Plasmid Construction

**Figure 1:**
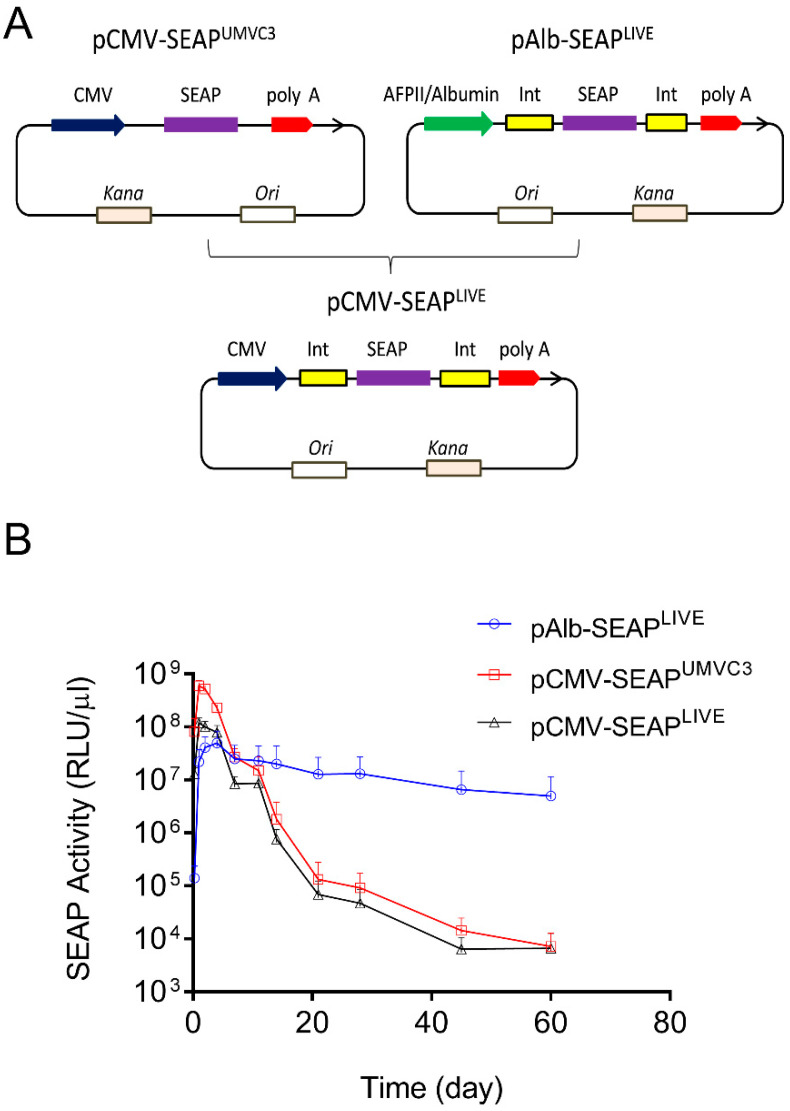
The promoter dictates the pattern of transgene expression in mice. (**A**) Schematic map of pCMV-SEAP^UMVC3^, pAlb-SEAP^LIVE^, and pCMV-SEAP^LIVE^ plasmids. (**B**) Serum levels of SEAP activity in mice hydrodynamically transfected with pCMV-SEAP^UMVC3^, pAlb-SEAP^LIVE^, or pCMV-SEAP^LIVE^ plasmids. The serum of untransfected mice was used as a blank control in ELISA. The data presented are the mean ± SD (*n* = 5).

Two sets of plasmids were used in the study, one containing SEAP and the other containing the mIL10 gene. The pLIVE-SEAP (renamed as pAlb-SEAP^LIVE^) plasmid was purchased from Mirus Bio LLC (Product No. MIR5320). This plasmid, which has a size of 4992 bp, contains an albumin promoter. The plasmid map and DNA sequence are available on the company website (https://www.mirusbio.com/?s=pLIVE-SEAP, accessed on 10 April 2024). To generate pCMV-SEAP^LIVE^, the Alb promoter in pAlb-SEAP^LIVE^ was replaced with a CMV promoter made by PCR using the pUMVC3-mIL10 plasmid as the template. It should be noted that pAlb-SEAP^LIVE^ and pCMV-SEAP^LIVE^ have the exact same plasmid backbone (Figure 1A).

The SEAP gene in both pAlb-SEAP^LIVE^ and pCMV-SEAP^LIVE^ was replaced with the mIL10 gene, which was PCR amplified from pUMVC3-mIL10 to generate pAlb-mIL10^LIVE^ and pCMV-mIL10^LIVE^ plasmids. For the construction of plasmids with different segments of the CMV promoter, PCR amplification was used to generate the needed fragments, which were then inserted into pAlb-SEAP^LIVE^ and pAlb-mIL10^LIVE^ plasmids at BglII and KpnI sites. Fragments containing different transcription factor binding sites (−463 > +1, −193 > +1, −103 > +1, and −93 > +1 = NoTFBS) of the CMV promoter were PCR generated using primers listed in Table S1. They were cloned into pCMV-mIL10^LIVE^ and pCMV-SEAP^LIVE^ plasmids to replace the CMV promoter, respectively. The different segments of the Alb promoter containing a single transcription factor binding site (HNF4α, CEBPA, or HNF1) were prepared by PCR and cloned into pNoTFBS-SEAP^LIVE^ plasmids that have only a core sequence of the CMV promoter (−93 > +1). Plasmids were amplified in E. coli, purified by CsCl-ethidium bromide density-gradient ultracentrifugation, and kept in 0.9% sodium chloride until use.

### 2.2. Biological Reagents

The phospha-light chemiluminescent SEAP assay kit was from Applied Biosystems (Foster City, CA, USA). The ELISA kits for mIL10 were from eBioscience (San Diego, CA, USA). TRIzol^®^ reagents for RNA extraction were from Life Technologies (Carlsbad, CA, USA). PCR primers were made and provided by Sigma-Aldrich (St. Louis, MO, USA). Acetylhistone H3 and H4 Antibody Sampler Kits, including anti-acetyl histone H3 (Lys9), anti-acetyl histone H3 (Lys18), anti-acetyl histone H3 (Lys27), anti-acetyl histone H4 (Lys8), anti-histone H3 (D1H2) XP^®^, anti-histone H3 (D2B12) XP^®^ (ChIP Formulated), anti-rabbit IgG, and anti-mouse IgG antibodies were purchased from Cell Signaling Technology (Danvers, MA, USA). The anti-GAPDH monoclonal antibody was purchased from Santa Cruz Biotechnology (Santa Cruz, CA, USA). The anti-TATA box-binding protein antibody (1TBP18) was from Abcam (Cambridge, MA, USA).

### 2.3. Hydrodynamic Gene Delivery in Mice

Plasmid DNA in saline was hydrodynamically injected via the tail vein in a volume equivalent to 9% of body weight in 4–7 s to CD-1 mice (female, 19–21 g, Charles River Laboratory, Wilmington, MA, USA) following the procedure previously described [22]. Each mouse was injected with a dose of 10 µg of plasmid DNA. Blood was collected from the tail vein at the indicated time after the injection, and serum was prepared and stored at −20 °C until use. All animal procedures and ethics rules were followed according to an animal protocol approved by the Institutional Animal Care and Use Committee at the University of Georgia (AUP# A29-014 07-008-A3).

### 2.4. SEAP Assay

The activity of SEAP in mouse serum was detected by SEAP assay using a phospha-light chemiluminescent reporter gene assay kit (Applied Biosystems, Waltham, MA, USA) following the instructions from the supplier. Serum samples were first diluted with dilution buffer and incubated for 30 min at 65 °C. Diluted samples (50 µL) were mixed with 50 µL assay buffer and incubated for 5 min at room temperature. Then, reaction buffer (50 µL) was added to the mixture and incubated for 20 min, and phospha-light chemiluminescent signals were measured for 0.5 sec using a luminometer (FlexStation 3, Molecular Device, LLC, San Jose, CA, USA). The SEAP level was presented as relative light units (RLU) detected per µL of mouse serum.

### 2.5. ELISA for mIL10

The serum concentration of mIL10 was determined by an ELISA using commercial kits (cat: 88-7104, eBioscience) as per the supplier’s instructions. Briefly, the mIL10 capture antibody was placed in each well of the 96-well plates and left overnight at 4 °C. Serum samples at different dilutions were added and incubated for 2 h. This was followed by an incubation with a biotinylated anti-mIL10 antibody for 1 h at room temperature. Avidin-horseradish peroxidase solution was added and incubated for 30 min. Substrate solution was added and incubated for 15 min before the reaction was stopped by the addition of sulfuric acid. The plate was chromatically read in an ELISA reader (DYNEX Technologies, Inc., Chantilly, VA, USA) at 450 nm. The concentration of mIL10 in serum was calculated based on a standard curve established in each plate using pure mIL10 proteins.

### 2.6. Real-Time PCR

After hydrodynamic gene transfer, liver samples were collected at specified time intervals for quantification of mRNA levels of transcription factors. Total RNA was extracted from the liver using TRIzol^®^ reagent (Life Technologies) as described in the manual. Reverse transcription was performed using the 1st Strand cDNA Synthesis System for Quantitative RT-PCR system (Origene, Rockville, MD, USA). The real-time PCR was carried out using PerfeCTa^®^ SYBR^®^Green Fast Mix (Quanta Biosciences, Beverly, MA, USA) in a StepOnePlus™ Real-Time PCR System (Life Technologies). The real-time PCR was carried out using the primer pairs listed in Table S1. The relative mRNA levels of target genes in animals were scaled to the mean of the relative mRNA level of the housekeeping gene GAPDH. The difference between the groups was compared and statistically analyzed by a Student’s *t*-test or One-way ANOVA, with *p* < 0.05 considered significant. Linear regression and correlation were also analyzed with Pearson correlation.

### 2.7. Chromatin Immunoprecipitation

Chromatin immunoprecipitation (ChIP) was performed using the chromatin immunoprecipitation assay kit from Millipore (Burlington, MA, USA), with some modifications. Fresh liver samples were collected after hydrodynamic injection and then cut into small pieces of approximately 1–3 mm^3^ in size using a razor blade on ice. These small pieces were cross-linked using 1.5% formaldehyde for 15 min at room temperature in 1 mL of PBS with a protease inhibitor cocktail from Sigma Aldrich (Burlington, MA, USA). To stop the cross-linking reaction, glycine was added at a final concentration of 0.125 M and incubated for 5 min at room temperature. The treated liver samples were washed twice with cold PBS for 5 min at 4 °C and resuspended in 1 mL of PBS containing a protease inhibitor cocktail. The samples were then disaggregated into a single-cell suspension using a Dounce homogenizer, and cells were pelleted by centrifugation at 300× *g* for 5 min at 4 °C. The cell pellet was resuspended in 200 µL of SDS lysis buffer (1% SDS, 50 mM Tris-Cl, 10 mM EDTA, pH 8.1) for 10 min on ice. The chromatin DNA in the cell lysate was sheared to lengths between 200 and 800 base pairs by the Qsonica Sonicator Q500 (Fisher Scientific) and centrifuged for 10 min at 13,000× *g* at 4 °C. The sonicated cell lysate (200 µL) was diluted to 2 mL using ChIP dilution buffer (0.01% SDS, 1.2 mM EDTA, 16.7 mM Tris-Cl, 1.1% Triton X-100, 167 mM NaCl, pH 8.1). A small amount of diluted cell supernatant (1%) was kept to quantify the amount of DNA by real-time PCR for the calculation of the total input value. A supernatant of centrifuged samples (2 mL) was added and incubated with 80 µL of salmon sperm DNA/protein A agarose-50% slurry (Millipore) for 30 min at 4 °C to minimize the nonspecific background. The mixture was centrifuged at 2000 rpm at 4 °C, and the supernatant was collected and incubated with ChIP-grade anti-acetyl histone H3, anti-acetyl histone H4, and anti-histone H3 antibodies, respectively, at 4 °C overnight. Normal rabbit IgG (Cell Signaling) was used as a negative control. Salmon sperm DNA/protein A agarose-50% slurry (60 µL) was added to the mixture and incubated for 1 h at 4 °C for antibody/histone complexes to form. The agarose was pelleted and washed for 5 min at 4 °C with different buffers in the order of low salt buffer (0.1% SDS, 2 mM EDTA, 20 mM Tris-Cl, 1% Triton X-100, 150 mM NaCl, pH 8.1), high salt buffer (0.1% SDS, 2 mM EDTA, 20 mM Tris-Cl, 1% Triton X-100, 500 mM NaCl, pH 8.1), LiCl buffer (0.25 M LiCl, 1 mM EDTA, 10 mM Tris-Cl, 1% NP-40, 1% deoxycholate, pH 8.1), and TE buffer (1 mM EDTA, 10 mM Tris-Cl, pH 8.0), respectively. The antibody/histone/DNA complexes were eluted with 500 µL elution buffer (1% SDS, 0.1 M NaHCO_3_). The elute was incubated with 20 µL of 5 M NaCl to reverse the histone/DNA crosslinks at 65 °C for 4 h. Then the elute was incubated with 10 µL 0.5 M EDTA, 20 µL 1 M Tris-Cl, and proteinase K for 1 h at 45 °C. DNA was extracted by phenol/chloroform, and the target DNA sequences were quantitated by real-time PCR in a StepOnePlus™ Real-Time PCR System (Life Technologies). Quantification of promoter and SEAP cDNA regions in plasmids was carried out by real-time PCR using primer sequences listed in Table S1. The amount of immunoprecipitated DNA was calculated by the following equation to normalize a sample to the amount of chromatin added to each ChIP: (DNAIP-antibody—DNAIP-IgG)/DNAInput, where DNAInput was the total input of DNA described above; DNAIP-antibody was the amount of DNA pulled down by antibody; and DNAIP-IgG was the amount of DNA pulled down by normal IgG. Results from individual samples were presented as a percentage of input (%). The difference between different plasmids was compared and statistically analyzed by a Student’s *t*-test, with *p* < 0.05 considered significant.

### 2.8. Bioinformatical Analysis

The following steps were taken for bioinformatical analysis. Transcription factor binding sites in CMV and Alb promoters were obtained from the Encyclopedia of DNA Elements (ENCODE) consortium portal at the UCSC genome browser (http://genome.ucsc.edu/ENCODE/downloads.html, accessed on 10 April 2024). RefGenes were downloaded from the UCSC genome browser (http://genome.ucsc.edu, accessed on 10 April 2024). All the data was in the hg19 assembly. The promoter/enhancer sequence in the CMV and Alb promoter regions was analyzed by customer software in the C programming language.

### 2.9. Statistical Analysis

All statistical analysis was performed using the Student’s *t*-test. Data are presented as mean  ±  SEM or mean ± SD. *p*  <  0.05 was considered statistical significance.

## 3. Results

### 3.1. Promoter Dictates the Pattern of Transgene Expression in Mice

Two plasmids were employed in this study: the pCMV-SEAP^UMVC3^ plasmid with a pUMVC3 backbone and the pAlb-SEAP^LIVE^ plasmid with a pLIVE backbone [23,24]. Both plasmids contain the cDNA of the human secreted embryonic alkaline phosphatase (SEAP) reporter gene, but a different promoter drives SEAP expression (Figure 1A). The aim of this experiment was to determine whether the backbone sequence of the plasmids affects the pattern of SEAP expression. A new plasmid (pCMV-SEAP^LIVE^) was created, replacing the Alb promoter in pAlb-SEAP^LIVE^ with a CMV promoter cloned from the pCMV-SEAP^UMVC3^ plasmid. The pAlb-SEAP^LIVE^ and pCMV-SEAP^LIVE^ vectors have an identical DNA sequence except for a different promoter.

The plasmids were hydrodynamically injected into mice, and serum levels of SEAP protein were examined over time. Results in Figure 1B show that SEAP expression driven by a CMV promoter in pCMV-SEAP^UMVC3^ or pCMV-SEAP^LIVE^ plasmids reached a peak level at 6 × 10^8^ relative light units per µL of serum (RLU/µL) on day 1, followed by a 4600-fold decrease in 3 weeks. In contrast, SEAP levels in mice injected with the pAlb-SEAP^LIVE^ plasmid peaked at around 4 × 10^7^ RLU/µL on day 2 and sustained with time. Two months after gene transfer, the SEAP level in pAlb-SEAP^LIVE^ injected animals was approximately 1000-fold higher than that of animals injected with either pCMV-SEA^PUMVC3^ or pCMV-SEAP^LIVE^ plasmids. These results suggest that the pattern of transgene expression is determined by the promoter, and the backbones of the two employed plasmids do not seem to play an important role. The pLIVE plasmid backbone was used for constructing new plasmids containing different transcription factor binding sites.

### 3.2. Transcription Factor Binding Sites in CMV Promoter Are Important for Promoter Strength but Not in Favor of Prolonging Transgene Expression

**Figure 2:**
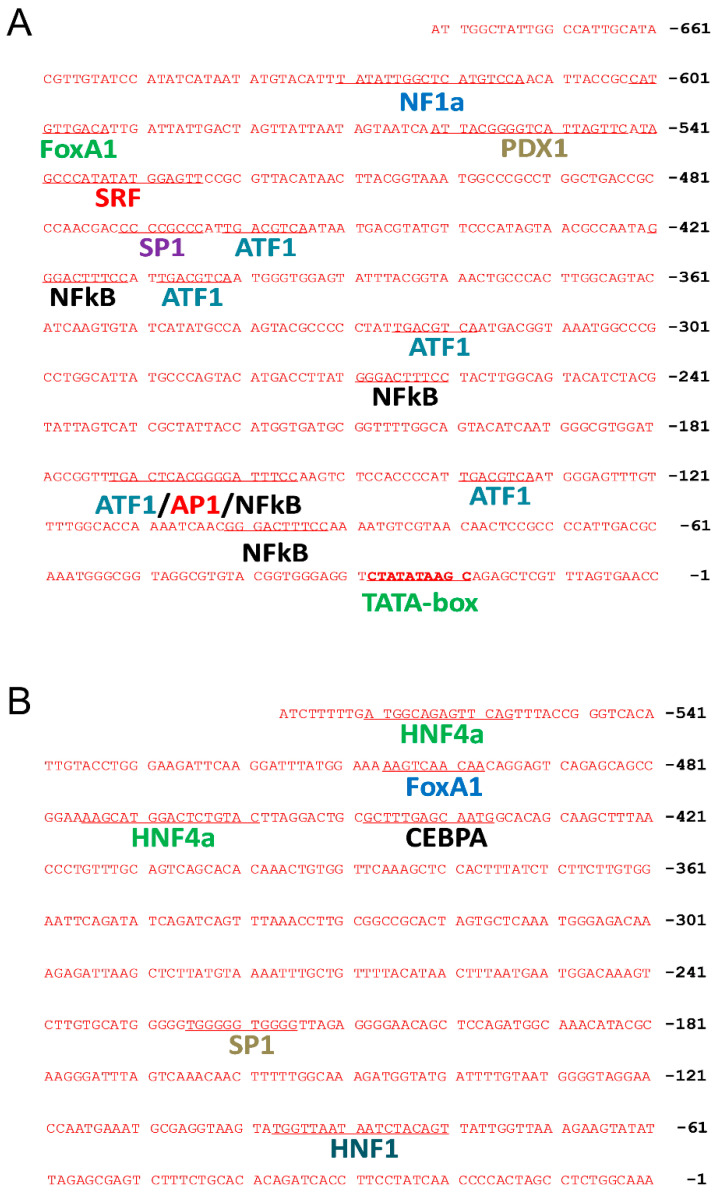
Computational prediction of transcription factor binding sites in CMV and the albumin promoter. Potential locations and sequences of transcription factor binding sites in CMV (**A**) and albumin (**B**) promoter regions were analyzed by computational methods.

The elements in the CMV promoter were analyzed to define their role in affecting transgene expression. The computer-based sequence analysis revealed 15 transcription factor binding sites in the CMV promoter (Figure 2), including 1 site for nuclear factor 1a (NF1a), 1 for forkhead box protein A1 (FOXA1), 1 for pancreatic and duodenal homeobox 1 (PDX1), 1 for serum response factor (SRF), 1 for transcription factor Sp1 (SP1), 5 for cyclic AMP-dependent transcription factor (ATF1), 4 for nuclear factor kappa-light-chain-enhancer of activated B cells (NFκB), and 1 for activator protein 1 (AP1). In contrast, fewer transcription factor binding sites were found in the Alb promoter, including 2 binding sites for hepatocyte nuclear factor 4α (HNF4α), 1 for FOXA1, 1 for CCAAT/enhancer-binding protein alpha (CEBPA), 1 for SP1, and 1 for hepatocyte nuclear factor 1 (HNF1).

**Figure 3:**
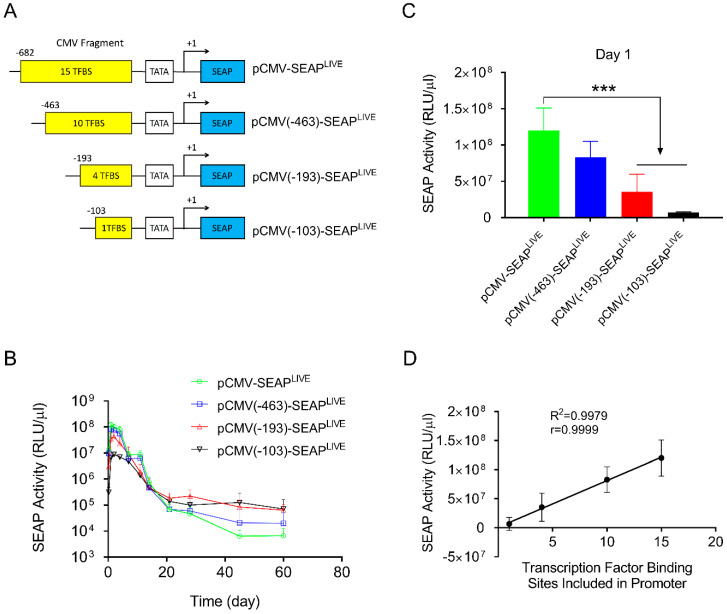
Transcription factor binding sites in CMV promoters dictate promoter strength. (**A**) Schematic presentation of CMV promoters with part of the promoter sequence deleted. (**B**) Serum levels of SEAP activity in mice injected with different plasmids. (**C**) Peak serum levels of SEAP activity 1 day after hydrodynamic injection. (**D**) Linear correlation between the number of transcription factor binding sites and the peak level of SEAP expression in mice (Pearson’s linear regression R^2^ = 0.9979, Pearson’s correlation r = 0.9999). The data presented are the mean ± SD (*n* = 5). ***, Student’s *t*-test, *p* < 0.001.

To reduce the number of transcription factor binding sites in the CMV promoter, three new plasmids were constructed. These included fragments of −463 > +1 containing 10 transcription factor binding sites (4 NFkB, 5 ATF1, 1 AP1); −193 > +1 containing 5 transcription factor binding sites (2 ATF1, 1 AP1, 2 NFkB); and −103 > +1 containing 1 NFkB binding site (Figure 3A). The plasmids were hydrodynamically transferred into mice, and SEAP expression was monitored and compared to a plasmid with a full sequence of the CMV promoter (−682 > +1). Results in Figure 3B show a similar pattern of SEAP gene expression for all 3 plasmid constructs tested, reaching a peak level 1 day after gene transfer, followed by a rapid decline thereafter. The peak level of SEAP activity in the −682 > +1 plasmid was 3.8- and 18-fold higher than that of plasmids containing −193 > +1 or −103 > +1 promoter fragments, respectively (Figure 3C, Student’s *t*-test *p* < 0.001). One-way ANOVA analysis also showed significant differences among the means of these 4 groups (*p* < 0.0001, F = 24.73). SEAP levels 60 days after gene delivery are higher in animals injected with plasmids with a shorter CMV promoter. Serum SEAP levels with promoter sequences of −193 > +1 and −103 > +1 are 10 and 11-fold higher than those of full CMV promoters (−682 > +1), respectively. The results suggest that transcription factor binding sites in the CMV promoter determine promoter strength but do not affect the pattern of transgene expression. The peak level of SEAP is directly proportional to the number of transcription factor binding sites in the CMV promoter (Figure 3D, Linear regression R^2^ = 0.9979, Pearson’s correlation r = 0.999).

### 3.3. Transcription Factor Binding Sites in Albumin Promoters Sustain Transgene Expression

**Figure 4:**
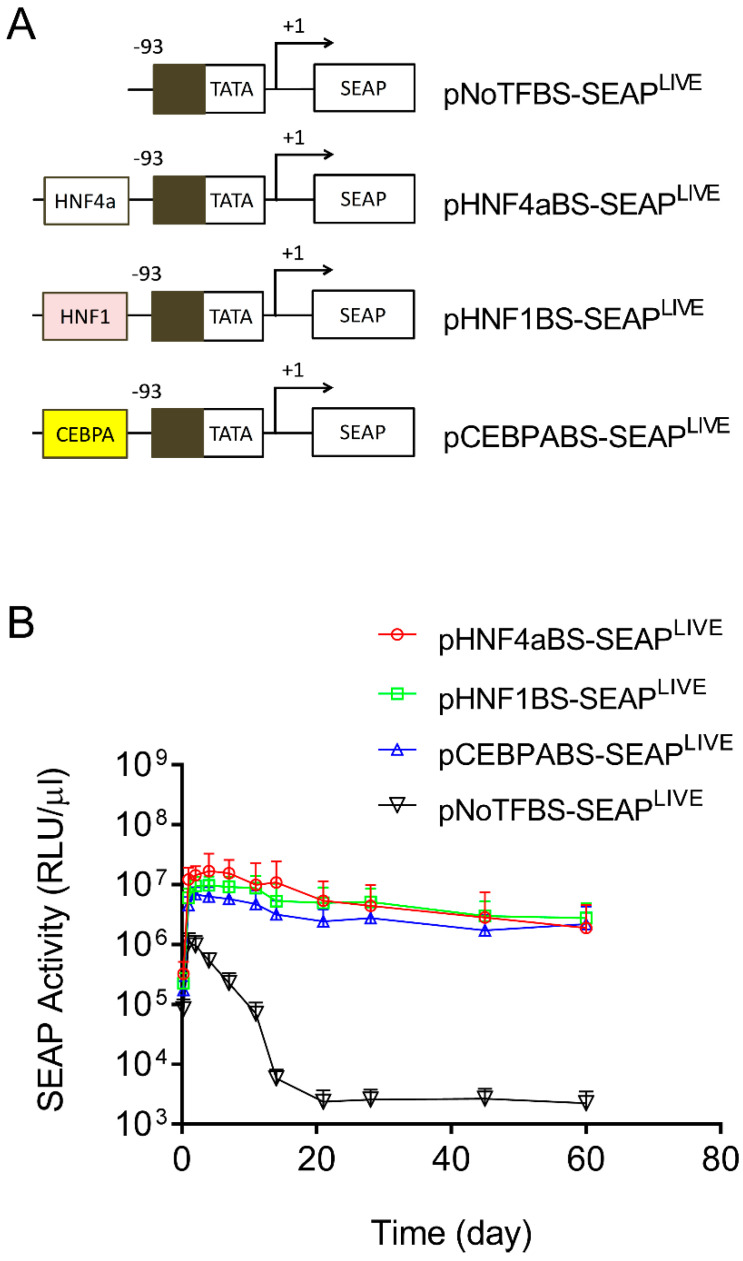
Transcription factor binding sites in albumin promoters sustain transgene expression. (**A**) Schematic presentations of promoters with selected binding sites for HNF4α, HNF1, or CEBPA. (**B**) Serum levels of SEAP in mice hydrodynamically injected with individual types of plasmids. The data presented are the mean ± SD (*n* = 5).

In addition to the above effort to identify the elements in the CMV promoter responsible for promoter strength and patterns of transgene expression, we made a similar effort to identify sequences in the Alb promoter that are responsible for sustaining transgene expression. Toward this end, we first created a plasmid with all transcription factor binding sites deleted in the promoter region of pCMV-SEAP^LIVE^. As shown in Figure 4A, the pNoTFBS-SEAP^LIVE^ plasmid (NoTFBS: no transcription factor binding site) has only a core CMV promoter sequence (−93 > +1). We then cloned the individual transcription factor binding site from the Alb promoter for HNF4a, HNF1, or CEBPA to the 5′ end of the TATA box region of the pNoTFBS-SEAP^LIVE^ plasmid. The hydrodynamic gene transfer procedure was performed with each of the new plasmid constructs, and the patterns of SEAP expression in transfected animals were examined. Results in Figure 4B show a rapid decline of SEAP expression from the promoter-less plasmid (pNoTFBS-SEAP^LIVE^). Plasmids containing elements that recognize HNF4a (pHNF4aBS-SEAP^LIVE^), HNF1 (pHNF1BS-SEAP^LIVE^), or CEBPA (pCEBPABS-SEAP^LIVE^) show sustained SEAP expression with a slight difference in promoter strength. The order of promoter strength judged by peak SEAP levels is HNF4a > HNF1 > CEBPA > ATF1 > NoTFBS. The SEAP expression patterns of plasmids containing transcription factor binding sites of the Alb promoter are comparable to those of pAlb-SEAP^LIVE^, as shown in Figure 1B.

### 3.4. Verification of the Activity of Promoter Sequences Using a Different Reporter Gene

**Figure 5:**
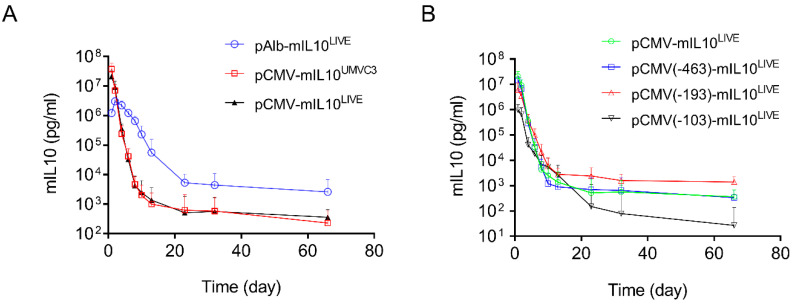

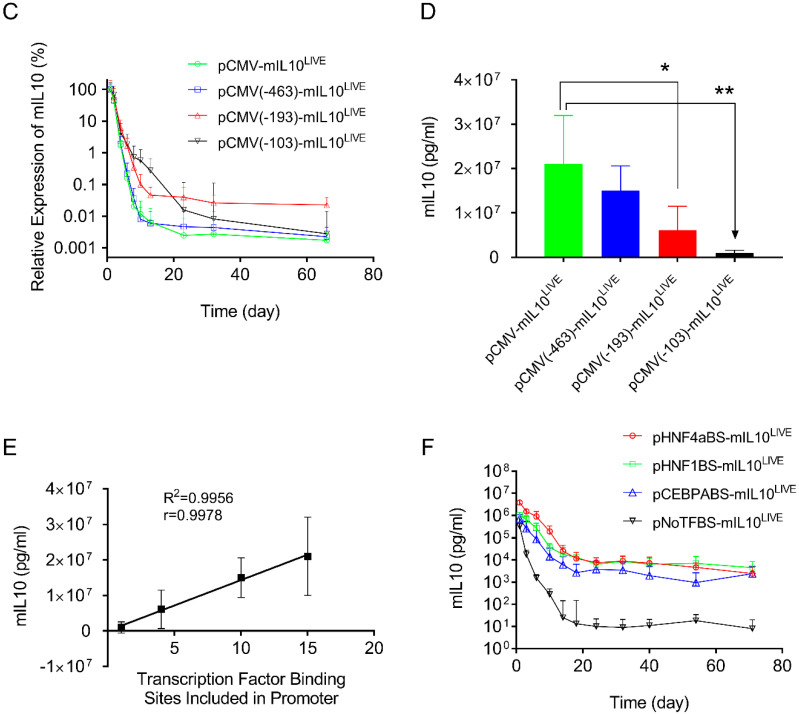
Effects of different promoters on serum levels of mIL10 in mice after hydrodynamic plasmid transfer. (**A**) Effects of the full promoter sequence between CMV and albumin on levels and persistency of mIL10 expression. (**B**) Effects of the length of the CMV promoter on the pattern of mIL10 expression. (**C**) Relative mIL10 expression in B. The peak concentration of mIL10 on day 1 was considered to be 100% in each plasmid. The concentrations of mIL10 in samples collected subsequently were normalized against it. (**D**) Peak serum levels of mIL10 protein 1 day after hydrodynamic injection. (**E**) Linear correlation between the number of transcription factor binding sites and the peak level of mIL10 expression in mice (Pearson’s linear regression R^2^ = 0.9956, Pearson’s correlation r = 0.9978). (**F**) Effects of individual transcription factor binding sites in the promoter on patterns of mIL10 gene expression. The data presented are the mean ± SD (*n* = 5). *, Student’s *t*-test *p* < 0.05; **, Student’s *t*-test *p* < 0.01.

The above results clearly show that two types of transcription factor binding sites exist in promoters, one capable of enhancing promoter strength (the transcription factor binding sites in the CMV promoter, Figure 3B) and the other prolonging transgene expression in mouse liver (the transcription factor binding sites from the albumin promoter, Figure 4B). A different reporter, the mouse mIL10 gene, was employed to verify such an observation. Compared to the long blood half-life of SEAP protein (7 days) [24], the blood half-life of mIL10 protein is significantly shorter (60 min) [25]. A series of plasmid constructs were made by cloning the cDNA of the mIL10 gene into the plasmid vectors that have been characterized for SEAP expression. The patterns of mIL10 gene expression resulting from new plasmid constructs were examined. Figure 5A shows the typical 2-phase kinetics of mIL10 protein level in blood: a rapid decline phase followed by a much slower phase starting approximately 20 days after hydrodynamic gene delivery. pAlb-mIL10^LIVE^ plasmids showed a significantly slower decline and a higher level of mIL10 protein in the second phase than plasmids driven by a CMV promoter, including pCMV-mIL10^UMVC3^ and pCMV-mIL10^LIVE^. Results in Figure 5B,C show the effects of shortened CMV promoters on the patterns of mIL10 expression. An identical and rapid decrease in the blood level of mIL10 protein was seen in transfected animals injected with plasmids containing a shorter CMV promoter. The peak level of mIL10 in the −682 > +1 plasmid was 3.4- and 22-fold higher than that of plasmids containing −193 > +1 or −103 > +1 promoter fragments, respectively (Figure 5D, Student’s *t*-test *p* < 0.05). One-way ANOVA analysis also showed significant differences among the means of these 4 groups (*p* = 0.0011). The peak level of mIL10 is also directly proportional to the number of transcription factor binding sites in the CMV promoter (Figure 5E, Linear regression R^2^ = 0.9956, Pearson’s correlation r = 0.9978). The effects of a single transcription binding site inserted into the 5′-end of the TATA box region of the pNoTFBS-mIL10^LIVE^ plasmid are shown in Figure 5F. It is evidenced that plasmids with transcription factor binding sites inserted into the promoter region of the pNoTFBS-mIL10^LIVE^ vector for HNF4a, HNF1, and CEBRA resulted in patterns of mIL10 expression almost identical to those of the pAlb-mIL10^LIVE^ plasmids. These results are in full agreement with those plasmids containing the SEAP reporter.

### 3.5. Impact of Hydrodynamic Injection on the Expression of Transcription Factor Genes

**Figure 6:**
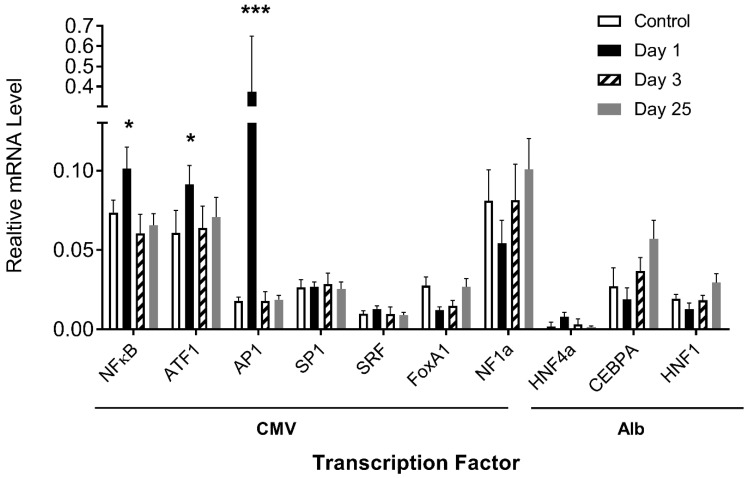
Effects of hydrodynamic gene transfer on mRNA levels of transcription factors in mouse liver. The mRNA levels of a transcription factor with binding sites in CMV and albumin promoters were determined in the livers of mice with or without (control) hydrodynamic gene transfer. The relative mRNA levels of transcription factor genes were scaled to those of the GAPDH level. The data presented are the mean ± SD (*n* = 3). *, *p* < 0.05; ***, *p* < 0.001.

The above results demonstrate that transcription factor binding sites play an important role in determining the pattern of transgene expression. We then examined whether the procedure used in the study would elevate the expression of transcription factors in the liver. Quantitative PCR (Q-PCR) was performed using the total mRNAs extracted from mouse livers on days 1, 3, and 25 after plasmid DNA injection and probed with primers recognizing the coding sequence of a specific transcription factor gene. Results in Figure 6 show a lack of significant increase in mRNA levels, compared to the control, of almost all transfection factor genes except AP1, ATF1, and NFκB, exhibiting 21-, 1.5-, and 1.4-fold increases on day 1 after hydrodynamic gene transfer, respectively, which returned to normal levels on day 3. There is no clear co-relationship observed between the mRNA level of transcription factor genes and the pattern of reporter gene expression, suggesting that the transcription factor level did not play an important role in determining the pattern of reporter gene expression.

### 3.6. Sequence Dependent Binding of Acetylated Histones to the Promoter

**Figure 7:**
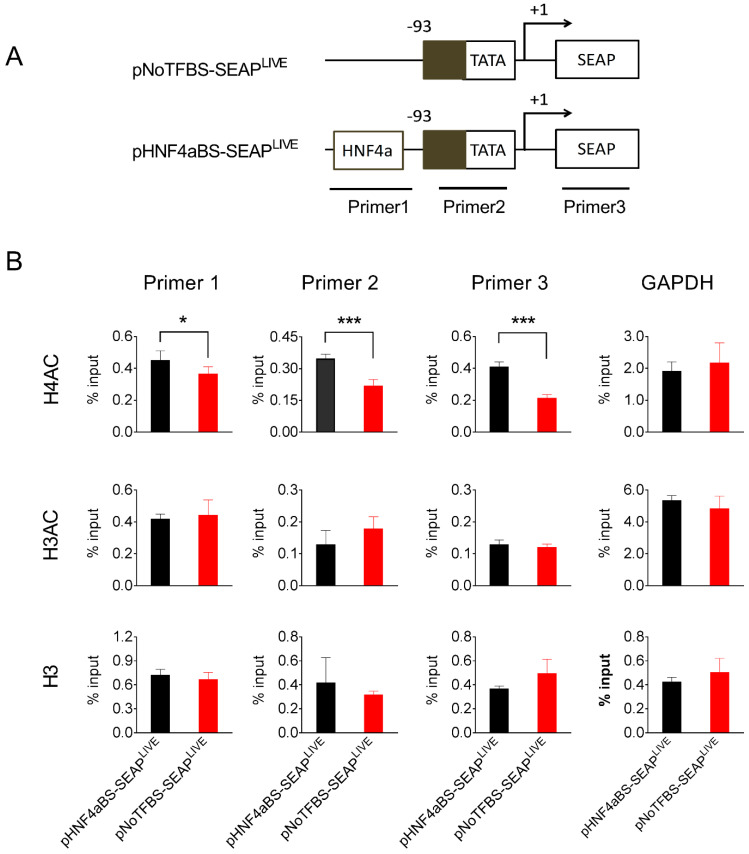
The HNF4α binding site enhances acetylated histone binding to the promoter. (**A**) Schematic presentation of pNoTFBS-SEAP^LIVE^ and pHNF4aBS-SEAP^LIVE^ plasmids and the PCR amplification sites in the ChIP assay. (**B**) The relative PCR product levels obtained with different primers from the immunocomplexes precipitated by antibodies against H4AC, H3AC, or H3. The PCR product level obtained from primers recognizing the GAPDH promoter region was used as the positive control. Mouse liver lysates were collected 25 days after hydrodynamic injection. The data presented are the mean ± SD (*n* = 4). *, *p* < 0.05; ***, *p* < 0.001.

To explore the possible mechanisms underlying the persistent transgene expression resulting from plasmids containing specific transcription factor binding sites, we chose pHNF4aBS-SEAP^LIVE^ plasmids, which drive sustained SEAP expression, and pNoTFBS-SEAP^LIVE^ plasmids, which generate the most transient SEAP expression, and studied their binding to different forms of histones in the mouse liver after hydrodynamic plasmid delivery. Liver samples were collected 25 days after plasmid transfer of either pNoTFBS-SEAP^LIVE^ or pNHF4aBS-SEAP^LIVE^, and a chromatin immunoprecipitation (ChIP) assay with antibodies against different histone moieties was performed. Immunocomplexes precipitated by histone antibodies were analyzed for the presence of fragments of plasmid DNA using PCR with primers recognizing two promoter regions or the SEAP region (Figure 7A). Results in Figure 7B show that binding of the euchromatin marker H4AC (acetylated on lysine residues 5, 8, 12, and 16 of histone H4) to the promoter region and SEAP regions in the pHNF4αBS-SEAP^LIVE^ plasmid is 2-fold higher than that of the pNoTFBS-SEAP^LIVE^ plasmid. Enrichment of euchromatin marker H3AC (acetylated on lysine residues 9 and 14 of histone H3) on HNF4α and No-TF plasmids was similar. There seemed to be more histone H3 protein binding to the pNoTFBS-SEAP^LIVE^ plasmid than the pHNF4αBS-SEAP^LIVE^ plasmid, although the difference was not statistically significant. As expected, there was no difference in the enrichments of histone proteins (H4AC, H3AC, and H3) in the GAPDH promoter region (Figure 7B). Compared to the pNoTFBS-SEAP^LIVE^ plasmid, significantly higher enrichment of H4AC and less enrichment of H3 on pHNF4αBS-SEAP^LIVE^ plasmids suggests that the persistence of transgene expression from pHNF4αBS-SEAP^LIVE^ plasmids is due to a high level of acetylated histones (H4AC) associated with the promoter region of this plasmid.

### 3.7. HNF4a Binding Sequence Enhances the Binding of TATA Box Binding Protein to the Promoter

**Figure 8:**
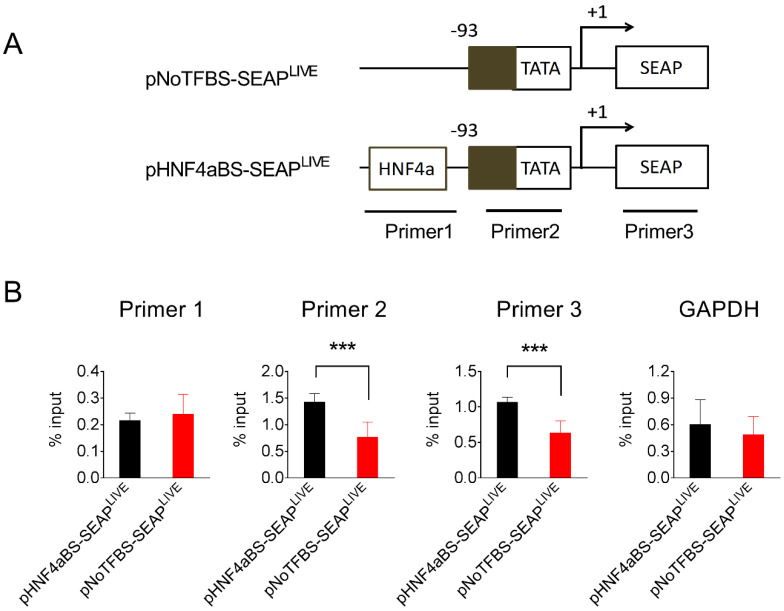
The HNF4α binding site increased the binding of the TATA box-binding protein to the promoter region. (**A**) Schematic presentation of pNoTFBS-SEAP^LIVE^ and pHNF4aBS-SEAP^LIVE^ plasmids and the promoter region for PCR amplification. (**B**) The relative PCR product levels obtained from the immunocomplexes precipitated by antibodies against TATA-binding protein. The PCR product level against the GAPDH promoter serves as a positive control. The data presented are the mean ± SD (*n* = 4). ***, *p* < 0.001.

TATA-box binding protein (TBP) is a core subunit of the pre-initiation complex of transcription [26,27,28]. The binding of TBP to promoters is required for transcription initiation by RNA polymerase II in eukaryotes [29]. Data in Figure 7 show that the HNF4α binding site recruits acetylated histones to the promoter region. We further investigated whether the HNF4α binding site affects the binding of TBP to the promoter region. pHNF4αBS-SEAP^LIVE^ and pNoTBSF-SEAP^LIVE^ plasmids were transferred into mice by hydrodynamic injection, and TBP binding to the promoter region of the injected plasmids was detected by a ChIP assay using anti-TBP antibodies in the mouse liver 25 days after injection. More PCR products to the TATA box and SEAP regions were detected in the anti-TBP pull-down complexes in mouse liver injected with pHNF4αBS-SEAP^LIVE^ plasmids (Figure 8). No difference was seen in the binding of TBP to the GAPDH promoter region in these two groups of animals. These results suggest that the HNF4α binding site in the pHNF4αBS-SEAP^LIVE^ plasmid is associated with a higher level of TBP.

## 4. Discussion

In this study, we demonstrate that transcription factor binding sites in a promoter are crucial in determining the pattern of transgene expression in mouse liver. The CMV promoter has a high number of transcription factor binding sites, making it strong but leading to a rapid decline in the level of gene product. In contrast, the albumin promoter has a lower number of transcription factor binding sites, resulting in lower but sustained transgene expression in mice. By substituting transcription factor binding sites in the CMV promoter with those of the albumin promoter and recognizing HNF4a, CEBPA, or HNF1, we were able to convert the pattern of transient transgene expression to sustained transgene expression. Our chromatin immunoprecipitation study revealed that persistent transgene expression is correlated with a higher level of association of acetylated histones and TATA box-binding protein to the promoter.

Results in Figure 1B and Figure 5A show that peak levels of SEAP and mIL10 expression are significantly higher when a CMV promoter drives reporter gene expression compared to the albumin promoter. The pattern of gene expression is highly predicted because the CMV promoter contains a significantly higher number of transcription factor binding sites. Each of which can bind to a transcription factor that enhances the binding of RNA polymerase II to the promoter to initiate transcription [30,31,32]. Our results in Figure 3A and Figure 5B show that the peak levels of SEAP and mIL10 are highest with a full CMV promoter and become lower when some transcription factor binding sites in the promoter are deleted. Plasmids with no transcription factor binding site in the promoter generated the lowest peak levels of SEAP and mIL10 (Figure 4B and Figure 5B).

One interesting observation made in our study is a transient increase in mRNA levels of the AP1, ATF1, and NFκB genes seen 1 day after hydrodynamic delivery (Figure 6). This increase is likely due to the physical nature of the procedure, which involves a transient disruption of cell membranes to facilitate gene transfer into cells. It is not clear whether the increase in mRNA levels of these genes would contribute to the peak and/or persistency of transgene expression. In principle, if the basal level of these transcription factors in hepatocytes is high enough for a maximal level of transgene expression, further increases of the same transcription factors will not make a difference because transgene expression is already at its peak. Otherwise, an increase in specific transcription factors could make a difference at an earlier time.

A sustained SEAP gene expression was seen in animals injected with pAlb-SEAP^LIVE^ (Figure 1B). However, a progressive decrease in serum levels of mIL10 (Figure 5B) was seen in animals injected with the same plasmid construct. The difference between the patterns of SEAP and mIL10 with albumin promoter is likely due to the shorter half-life of mIL10 protein in blood circulation, which is about 1 h [25], compared to 7 days for SEAP [33].

Our results suggest that transcription factor binding sites in the promoter are critical for sustaining transgene expression. Promoter sequences for HNF4a, HNF1, and CEBPA are the types of elements capable of sustaining transgene expression in the mouse liver. While additional work is needed to examine whether other transcription factor binding sites would also be able to drive sustained transgene expression, our results suggest that these hepatic transcription factors are capable of recruiting the acetyltransferase to the promoter region for histone modification. It is the acetylated histone in the promoter region that keeps the promoter accessible and transcription continues. Additional studies are needed to examine whether DNA methylation also plays a role under our experimental conditions.

Mechanistically, when plasmids reach the nucleus of hepatocytes after hydrodynamic gene delivery, plasmid DNA molecules will interact with nuclear proteins. Two types of interactions may take place: a sequence-specific binding of transcription factors to the promoter and an ionic binding of positively charged histones to plasmid DNA [21,27,34,35]. Kinetically, transcription factor binding to the promoter sequence is favored at the early time point due to sequence specificity and high binding affinity, resulting in the recruitment of RNA polymerase II and other associated proteins to the promoter region for transcription initiation [27,28,36]. It is likely that the ionic interaction between histones and plasmid DNA will dominate eventually due to histone abundance in the nucleus and the low dissociation between DNA and histone in the complexes, which is the case for CMV-driven expression. In principle, epigenetic modification through histone acetylation is the only way to separate DNA from histones and keep promoters accessible. Consequently, plasmid vectors capable of recruiting epigenetic modification to the promoter region, such as those plasmids containing albumin promoters or elements of albumin promoters, could lead to sustained transgene expression in the liver.

One critical finding in the current study is that a single transcription factor binding site in a promoter is sufficient to drive sustained transgene expression. Future studies are likely to reveal the properties of different transcription factor binding sites and their activities in driving transgene expression in different tissues. Additional studies are needed to determine how the relative location, the distances between, order, and the direction of the transcription factor binding sites in the promoter affect the pattern of transgene expression. The role of the transcription factor binding sites in determining the pattern of transgene expression in different types of cells also needs to be tested for optimal transgene expression. The influence of mRNA and protein half-lives and post-translational modification on the pattern of transgene product level needs additional work. Nevertheless, the results presented in this manuscript would encourage further investigation into new gene expression cassettes based on the sequences of transcription binding sites to satisfy various needs of gene therapy.

## 5. Conclusions

A pivotal discovery from our study is the potency of a single transcription factor binding site with a promoter in driving sustained transgene expression, while promoter strength appears to be determined by the number of transcription factor binding sites. The persistence of gene expression is determined by the presence of regulatory elements in the promoter capable of recruiting epigenetic-modifying complexes that affect promoter accessibility for transcription.

## Data Availability

The original contributions presented in the study are included in the article and Supplementary Materials, further inquiries can be directed to the corresponding author.

## Supplementary Materials

The following supporting information can be downloaded at: https://www.mdpi.com/article/10.3390/pharmaceutics16040544/s1, Table S1. Primer sets used in PCR-based amplification.
