# Supplementary material for: Transcription Factor Binding Site in Promoter Determines the Pattern of Plasmid-Based Transgene Expression In Vivo
Source: Pharmaceutics. 2024 Apr 15;16(4):544. doi: 10.3390/pharmaceutics16040544 (PMC11055139; doi:10.3390/pharmaceutics16040544)
Supplement: Supplementary file 1 [file pharmaceutics-16-00544-s001.zip › pharmaceutics-2884615-supplementary.pdf]

Table S1. Primer sets used in PCR-based amplification

Name	Forward sequence	Reverse sequence
1. Amplification of selected region of CMV promoter		
-463 > +1	5'-GGAAGATCTTTGACGTCAATAATGACGTAT-3'	5'-ACGGGGTACCTCACTCTTGGCACGGGGAAT-3’
-193 > +1	5'-GGAAGATCTAATGGG CGTGGATAGCGGTT-3'	5'-ACGGGGTACCTCACTCTTGGCACGGGGAAT-3’
-103 > +1	5'-GGAAGATCTCGGGACTTTCCAAAATGTCGT-3'	5'-ACGGGGTACCTCACTCTTGGCACGGGGAAT-3’
-93 > +1 (NoTFBS)	5'-GGAAGATCTCAAAATGTCGTAACAACTCC-3'	5'-ACGGGGTACCTCACTCTTGGCACGGGGAAT-3’
2. Amplification of DNA fragment with one transcription factor binding site of Alb promoter [a]		
HNF4α	5'-GGAAGATCTAGGTCAAAGTCCAGCAAAATGTCGTAACAACTCC-3'	5'-ACGGGGTACCTCACTCTTGGCACGGGGAAT-3’
CEBPA	5'-GGAAGATCTGCATTGCACAATGCCAAAATGTCGTAACAACTCC-3'	5'-ACGGGGTACCTCACTCTTGGCACGGGGAAT-3’
HNF1	5'-GGAAGATCT TGGTTAATAATCTACAG CAAAATGTCGTAACAACTCC-3'	5'-ACGGGGTACCTCACTCTTGGCACGGGGAAT-3’
3. Quantification of DNA sequences resulted from ChIP		
Promoter Region:	5'-GGAAGATCTCGGGACTTTCCAAAATGTCGT-3'	5'-GGTACCGGTTCACTAAA CGAGCTCTGCTTA-3'
SEAP Region:	5'-CTCCAACATGGACGCATTGACG-3'	5'-CCCATGCGAAACATGTACTTT-3'
GAPDH Promoter:	5'-CTGCAGTACTGTGGGGAGGT-3'	5'-CAAAGGCGGAGTTACCAGAG-3
4. Detection of mRNA level of transcription factor in the liver of hydrodynamically injected mouse		
NFκBp65	5'-AGGCTTCTGGGCCTTATGTG-3'	5'-TGCTTCTCTCGCCAGGAATAC-3'
ATF1	5'-GATTCCCACAAGAGTAACACGAC-3'	5'-CCTATGCTGTCAGATGAGTCCT-3'
AP1	5'-CCTTCTACGACGATGCCCTC-3'	5'-GGTTCAAGGTCATGCTCTGTTT-3
SP1	5'-GCCGCCTTTTCTCAGACTC-3'	5’-TTGGGTGACTCAATTCTGCTG-3'
SRF	5'-GGCCGCGTGAAGATCAAGAT-3'	5'-CACATGGCCTGTCTCACTGG-3'
FOXA1	5'-ATGAGAGCAACGACTGGAACA-3'	5'-TCATGGAGTTCATAGAGCCCA-3'
NF1α	5'-GCAGCCAGTCCAGTGGATG-3'	5'-TGCTCTGGGTCCTGTAATGAC-3'
HNF4α	5'-GTGGCGAGTCCTTATGACACG-3'	5'-GCTGTTGGATGAATTGAGGTTGG-3'
CEBPA	5'-CAAGAACAGCAACGAGTACCG-3'	5'-GTCACTGGTCAACTCCAGCAC-3'
HNF1	5'-GACCTGACCGAGTTGCCTAAT-3'	5'-CCGGCTCTTTCAGAATGGGT-3'
GAPDH	5'-AGGTCGGTGTGAACGGATTTG-3'	5'-TGTAGACCATGTAGTTGAGGTCA-3'

[a] The underlined sequences are the consensus sequences for the listed transcription factors. HNF4α: hepatocyte nuclear factor 4α; CEBPA: CCAAT/enhancer-binding protein α; HNF1: hepatocyte nuclear factor 1 homeobox A.
